# Validation of syncope short-term outcomes prediction by machine learning models in an Italian emergency department cohort

**DOI:** 10.1007/s11739-025-04034-x

**Published:** 2025-07-16

**Authors:** Alessandro Giaj Levra, Mauro Gatti, Roberto Mene, Dana Shiffer, Giorgio Costantino, Monica Solbiati, Raffaello Furlan, Franca Dipaola

**Affiliations:** 1 Department of Cardiovascular Medicine, Humanitas Research Hospital, IRCCS, Rozzano, Milan, Italy; 2 Department of Biomedical Sciences, Humanitas University, Pieve Emanuele, Milan, Italy; 3 IBM, Milan, Italy; 4 Roberto Mene Electrophysiology Unit, Niguarda Hospital, Milan, Italy; 5 Emergency Department, IRCCS Humanitas Research Hospital, Rozzano, Milan, Italy; 6 Emergency Department, Fondazione IRCCS Ca’ Granda Ospedale Maggiore Policlinico, Università degli Studi di Milano, Milan, Italy; 7 Internal Medicine, Syncope Unit, IRCCS. Humanitas Research Hospital, Via A. Manzoni, 56, Rozzano, 20089 Milan, Italy

**Keywords:** Artificial intelligence, Machine learning, Syncope, Risk prediction, Canadian Syncope Risk Score (CSRS), Gradient boosting, Large language models

## Abstract

Machine learning (ML) algorithms have the potential to enhance the prediction of adverse outcomes in patients with syncope. Recently, gradient boosting (GB) and logistic regression (LR) models have been applied to predict these outcomes following a syncope episode, using the Canadian Syncope Risk Score (CSRS) predictors. This study aims to externally validate these models and compare their performance with novel models. We included all consecutive non-low-risk patients evaluated in the emergency department for syncope between 2015 and 2017 at six Italian hospitals. The GB and LR models were trained and tested using previously validated CSRS predictors. Additionally, recently developed deep learning (TabPFN) and large language models (TabLLM) were validated on the same cohort. The area under the curve (AUC), Matthews correlation coefficient (MCC), and Brier score (BS) were compared for each model. A total of 257 patients were enrolled, with a median age of 71 years. Thirteen percent had adverse outcomes at 30 days. The GB model achieved the best performance, with an AUC of 0.78, an MCC of 0.36, and a BS of 0.42. Significant performance differences were observed compared with the TabPFN model (*p* < 0.01) and the TabLLM model (*p* = 0.01). The GB model performed only slightly better than the LR model. The predictive capability of the GB and LR models using CSRS variables was reduced when validated in an external syncope cohort characterized by a higher event rate.

## Introduction

Syncope is a common presentation in emergency departments (EDs) [1–3], with prognoses varying depending on the underlying cause, from benign vasovagal episodes to a significantly increased risk of sudden death in cardiac-related cases [2, 3]. Therefore, effective risk stratification in the ED is essential to promptly identify patients needing urgent evaluation, hospital admission, and appropriate treatment [4].

Over the past two decades, numerous clinical scores and decision rules have been developed to assist emergency physician in clinical decision making [5–8]. Among these, the San Francisco Syncope Rule (SFSR) [9], the Evaluation of Guidelines in Syncope Score (EGSYS) [10] and the Osservatorio Epidemiologico della Sincope nel Lazio (OESIL) [11] have demonstrated significant clinical potential in the ED due to their excellent negative predicting values. However, they have not proven superior to clinical judgment, limiting their implementation in clinical practice [12]. More recently, the Canadian Syncope Risk Score (CSRS) was developed as a clinical prediction score [7]. The CSRS is based on a regression model aimed at predicting 30-day occurrence of adverse events in patients with syncope, incorporating nine clinical predictors.

Artificial intelligence (AI) and machine learning (ML) have recently shown the capability to effectively predict the risk in patients with syncope [13–15]. A study by Grant et al. compared the effectiveness of several ML models in predicting adverse syncope outcomes against a more classical statistical approach, logistic regression (LR) [16]. Data from 11 Canadian EDs, previously used to validate the CSRS, provided a substantial dataset for training and testing these AI-based algorithms. Results suggested that certain ML models performed similarly to the original LR used for the CSRS but required fewer predictors. This reduced number of variables in ML-based predictive approaches is particularly advantageous in the ED, as it simplifies decision-making for emergency physicians.

These results suggest that novel ML techniques may improve prognostic prediction of syncope in the ED and outperform traditional risk stratification methods, thereby supporting more effective management.

Recent advances in ML have led to novel algorithms that may enhance prognostic prediction [17].

The objective of this study was to validate the ML models developed by Grant et al. in a cohort of syncope patients from EDs in Italy, a population with a distinct risk profile and healthcare system. Additionally, the study aimed to compare the performance of these models with that of novel AI algorithms.

## Methods

### Study design and population

The patients included in the current investigation were part of a larger prospective observational database encompassing 414 patients who presented with syncope at six Italian EDs between September 2015 and February 2017 [18].

The participating centers for recruitment were Humanitas Research Hospital (Milan, Italy), Niguarda Hospital (Milan, Italy), Luigi Sacco Hospital (Milan, Italy), Cà Granda Ospedale Maggiore Policlinico Hospital (Milan, Italy), Santa Croce Hospital (Moncalieri, Italy), and Santi Antonio e Biagio e Cesare Arrigo Hospital (Alessandria, Italy).

Of the original 414 patients, 257 were identified by the ED physicians as having a non-low-risk profile for short-term adverse events (as they underwent continuous ECG monitoring in the ED), meaning they were classified as intermediate and high risk. These 257 patients were subsequently followed up by phone after hospital discharge to identify adverse outcomes within 30 days. They constituted the study cohort of the current observational investigation. Exclusion criteria included transient loss of consciousness due to head trauma, association with alcohol or drug abuse, seizures, or secondary to acute conditions or disorders requiring acute therapeutic interventions (e.g. myocardial infarction, pulmonary embolism). Individuals unable to provide written informed consent, pregnant or breastfeeding were excluded from enrollment.

Variables analyzed in the study were collected upon ED admission. For each variable, only the first recorded value was considered if repeated measurements were available.

The present study complied with the Declaration of Helsinki and received approval from the institutional review board of the coordinating center (L. Sacco Hospital, approval number 608/2015). Written general informed consent, allowing the use of individual clinical data following anonymization, was obtained from all patients upon ED admission. All data were anonymized prior to processing.

Data is held in an adequate repository (Zenodo) and is available upon appropriate request to the corresponding author. Code is presently unavailable.

### Aims

We hypothesized that this selected subgroup of high-risk syncope patients represented the most challenging population to test the effectiveness of the ML models developed by Grant et al. for predicting short-term syncope outcomes. In addition, we hypothesized that evaluating these models in a healthcare system distinct from the Canadian context could further challenge their generalizability and efficacy.

### Definition

Adverse outcomes were defined according to the “Standardized reporting guidelines for emergency department syncope risk-stratification research” [19]. These included a composite of all-cause death, ventricular fibrillation, sustained and non-sustained symptomatic ventricular tachycardia, sinus arrest with cardiac pause greater than three seconds, sick sinus syndrome, second degree type 2 or third degree atrioventricular block, permanent pacemaker (PM) or implantable cardioverter defibrillator (ICD) malfunction with cardiac pauses, PM or ICD implantation, severe aortic stenosis (valve area < 1cm^2^), myocardial infarction, hypertrophic cardiomyopathy with outflow tract obstruction, left atrial myxoma or thrombus with outflow tract obstruction, pulmonary embolism, aortic dissection, occult haemorrhage or anaemia requiring transfusion, cardiopulmonary resuscitation, cerebrovascular events and recurrence of syncope resulting in major traumatic injury or hospital readmission [19]. Algorithms were trained to predict adverse events at thirty days.

### Models and predictors selection

The GB and LR models developed by Grant et al. were validated in the present study on an Italian cohort of patients who were seen in the ED for syncope. The GB model was selected as it achieved the highest AUC value in absolute terms. LR obtained high AUC scores using fewer predictors compared to the GB model. Additionally, two state-of-the-art ML models were implemented. The tabular Prior-Data Fitted Network (TabPFN), which is a deep learning (DL) model for tabular data, performing supervised classification tasks without requiring hyperparameter tuning. The second model was the Tabular Large Language Model (TabLLM), that is a Bidirectional Encoder Representations from Transformers (BERT) requiring the conversion of tabular data into a natural-language string. This latter model performs classification tasks based on brief user-provided descriptions [20–23].

Predictors of the GB, TabPFN and TabLLM models included the categorical and continuous variables previously used by Grant et al. The categorical variables included ED diagnosis of vasovagal syncope, troponin values greater than 99% of the normal population, ED diagnosis of cardiac syncope, history of heart disease, QRS duration greater than 130 ms, haemoglobin levels lower than 9 mg/dL, QTc greater than 480 ms, and abnormal QRS axis. Age was considered as a continuous variable (Table 1).

**Table 1:** Predictors previously implemented and used in the validation models

Gradient boosting, TabPFN, TabLLM	Logistic regression
Diagnosis of vasovagal syncope (categorical)	Diagnosis of vasovagal syncope (categorical)
Troponin values greater than 99% of the normal population (categorical)	Troponin values greater than 99% of the normal population (categorical)
Diagnosis of cardiac syncope (categorical)	Diagnosis of cardiac syncope (categorical)
Presence of heart disease history (categorical)	Presence of heart disease history (categorical)
QRS duration greater than 130 ms (categorical)	QRS duration greater than 130 ms (categorical)
QTc greater than 480 ms (categorical)	QTc greater than 480 ms (categorical)
Abnormal QRS axis (categorical)	Presence of bifascicular block(categorical)
Haemoglobin levels lowerethan 9 mg/dL (categorical)	
Age (numeric)	

*TabLLM* tabular large language model, *TabPFN* tabular prior-data fitted network, *QTc* corrected QT interval

Predictors of the LR model also consisted of previously validated variables, including ED diagnosis of vasovagal syncope, troponin values greater than 99% of the normal population, ED diagnosis of cardiac syncope, history of heart disease, QRS duration greater than 130 ms, haemoglobin levels greater than 9 mg/dL, QTc greater than 480 ms, and presence of bifascicular block (Table 1).

### Missing values

For each continuous variable, missing values were replaced with the mean value observed in the training set. Among the selected predictors, troponin had the highest rate of missing values (*n* = 158, 61%). Three different methods for troponin data imputation, namely the SimpleImputer, IterativeImpuiter and KNNInputer methods were tested. No significant differences were observed in the GB model’s performance. The remaining variables had fewer than 3% missing values.

### Outcomes and performance metrics

Models were trained to predict the occurrence of adverse outcomes using the training and validation sets. Algorithms predicting capability was evaluated using the following metrics: AUC, Brier score (BS) and Matthews correlation coefficient (MCC) [24, 25]. A paired Student’s *t* test was used on the MCC to determine statistically significant differences among models.

### Data processing

**Fig. 1:**
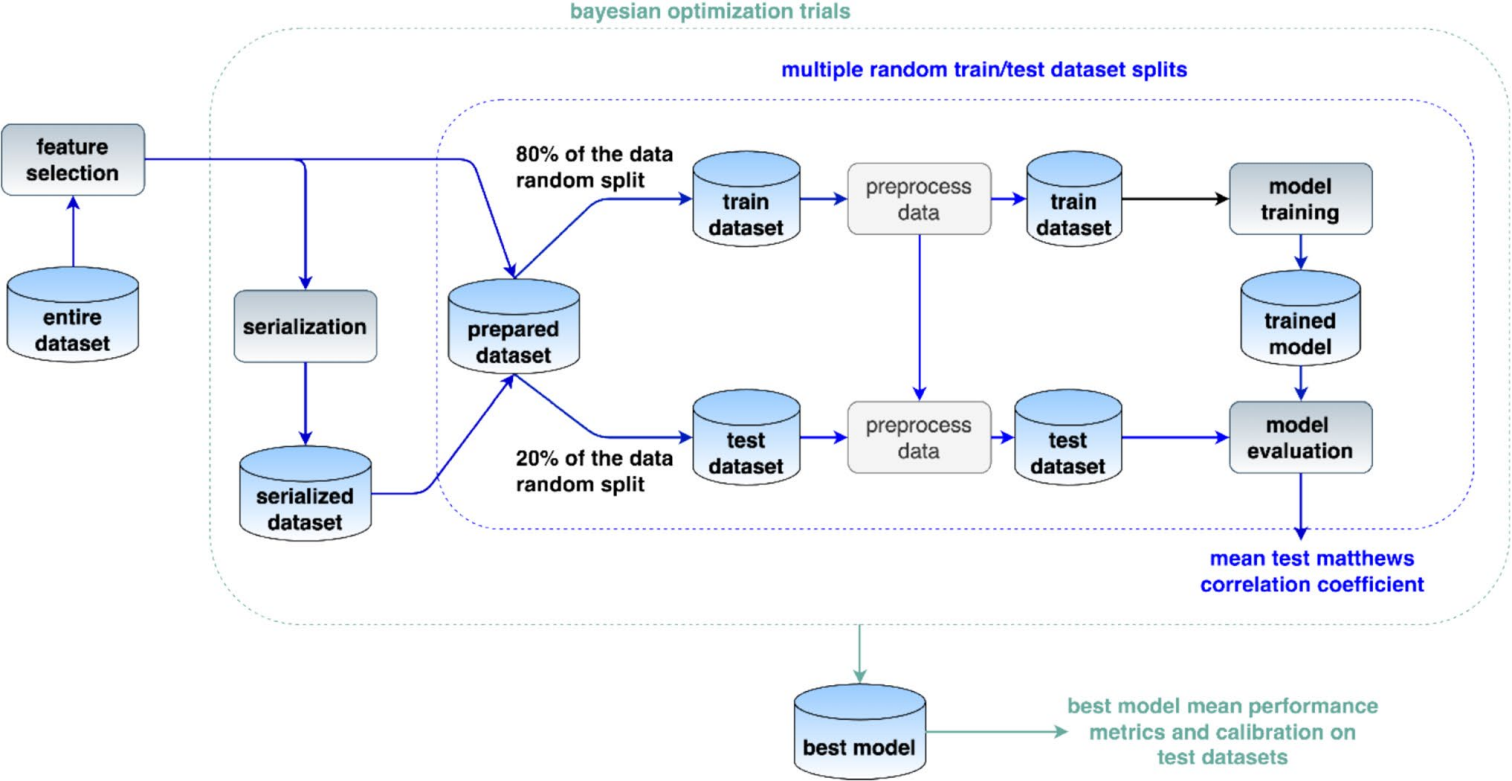
Data processing pipeline. Data are represented by cylinders, algorithms by rounded boxes. The arrows show the sequence of the elaborations with the associated dependencies. The blue dotted box contains a series of steps that are repeated multiple times with different random splits of the dataset into training and text data. The green dotted box contains multiple trials that are performed with different hyperparameters with the purpose of identifying the best hyperparameters by means of a Bayesian optimization

Initially, feature selection was conducted to identify candidate predictors. Only for the TabLLM model, features underwent serialization, converting tabular data into a textual format. The prepared dataset was subsequently split into a training set (80% of the original dataset) and a test set (20% of the original dataset). The split dataset was then pre-processed to handle the missing data points. Model training and evaluation were performed on 100 random splits of the original dataset. Hyperparameter tuning was carried out by a Bayesian optimization, meaning that hyperparameters were tuned to achieve the best mean MCC score on the test dataset over the multiple splits performed. Finally, model performance was assessed using the specified metrics along with calibration analysis. A comprehensive overview of the data processing pipeline is depicted in Fig. 1.

## Results

### Population

A total of 257 patients were enrolled, with a median age of 71 years (IQR: 51–81). An ED diagnosis of cardiac syncope was present in over three-quarters of the patients, and troponin values exceeded the 99th percentile of normal in 16% (*n* = 41). Thirty-day adverse events occurred in 13.2% (*n* = 34) of patients. Population demographic and clinical features are reported in Table 2.

**Table 2:** Population demographic and clinical features

Clinical features	Study dataset (*n* = 257)
Age, median	71 (51–81)
History of heart disease, *n* (%)	90 (35)
Troponin > 99th percentile, *n* (%)	41 (15.9)
Abnormal QRS axis, *n* (%)	39 (15.2)
QRS > 130 ms, *n* (%)	28 (10.9)
QT interval > 480 ms, *n* (%)	5 (1.9)
Diagnosis of vasovagal syncope, *n* (%)	61 (23.7)
Diagnosis of cardiac syncope, *n* (%)	196 (76.3)
Haemoglobin < 9, *n* (%)	8 (3.1)
Fascicular block, *n* (%)	10 (3.9)
Events at thirty days, *n* (%)	34 (13.2)

### Algorithms performance

**Fig. 2:**
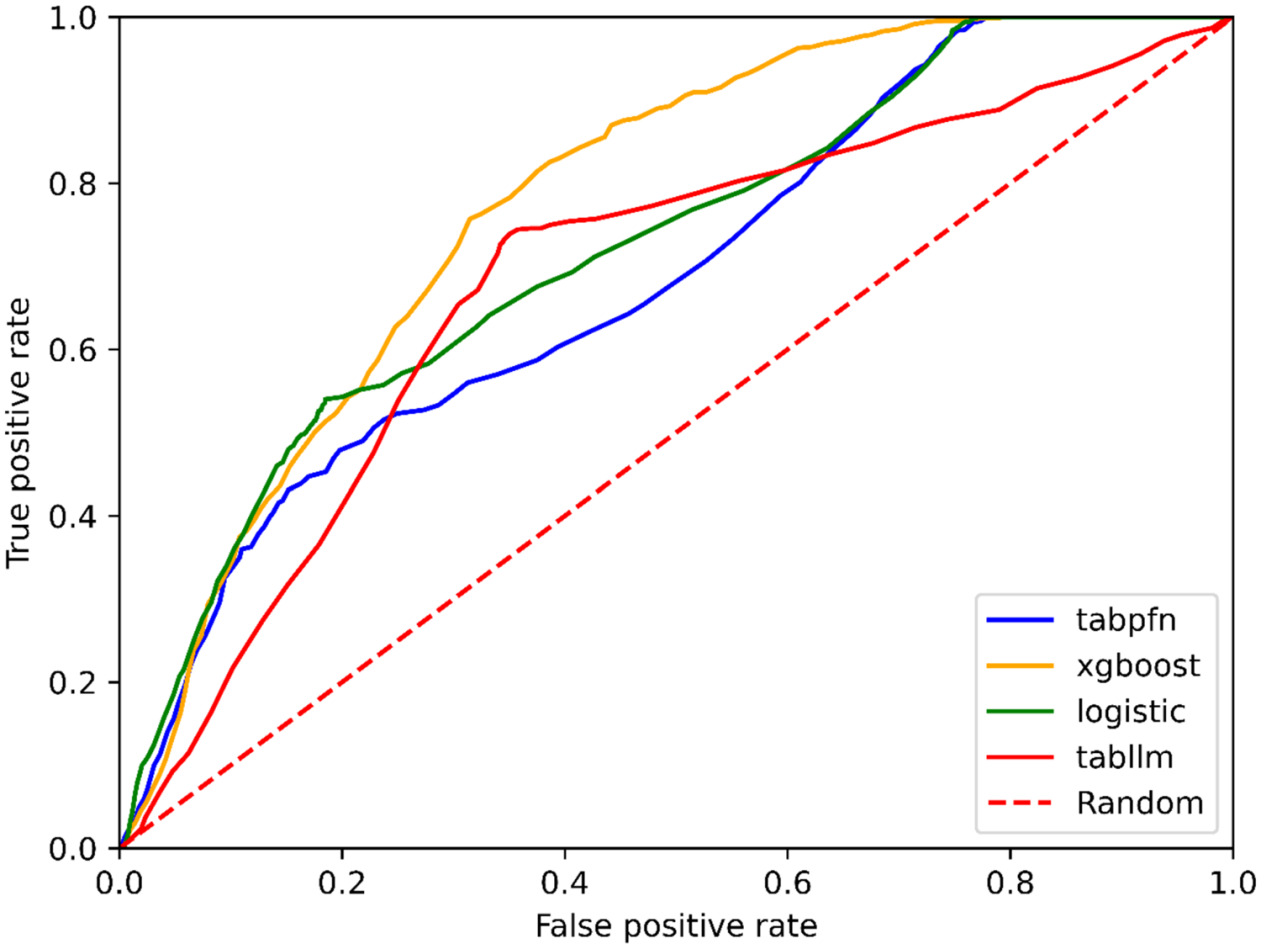
ROC curves of models. The red dotted line represents random predictions. The green line is the LR model. The red continuous line is the TabLLM model. The yellow line is the GB model. The blue line is the TabPFN model

**Fig. 3:**
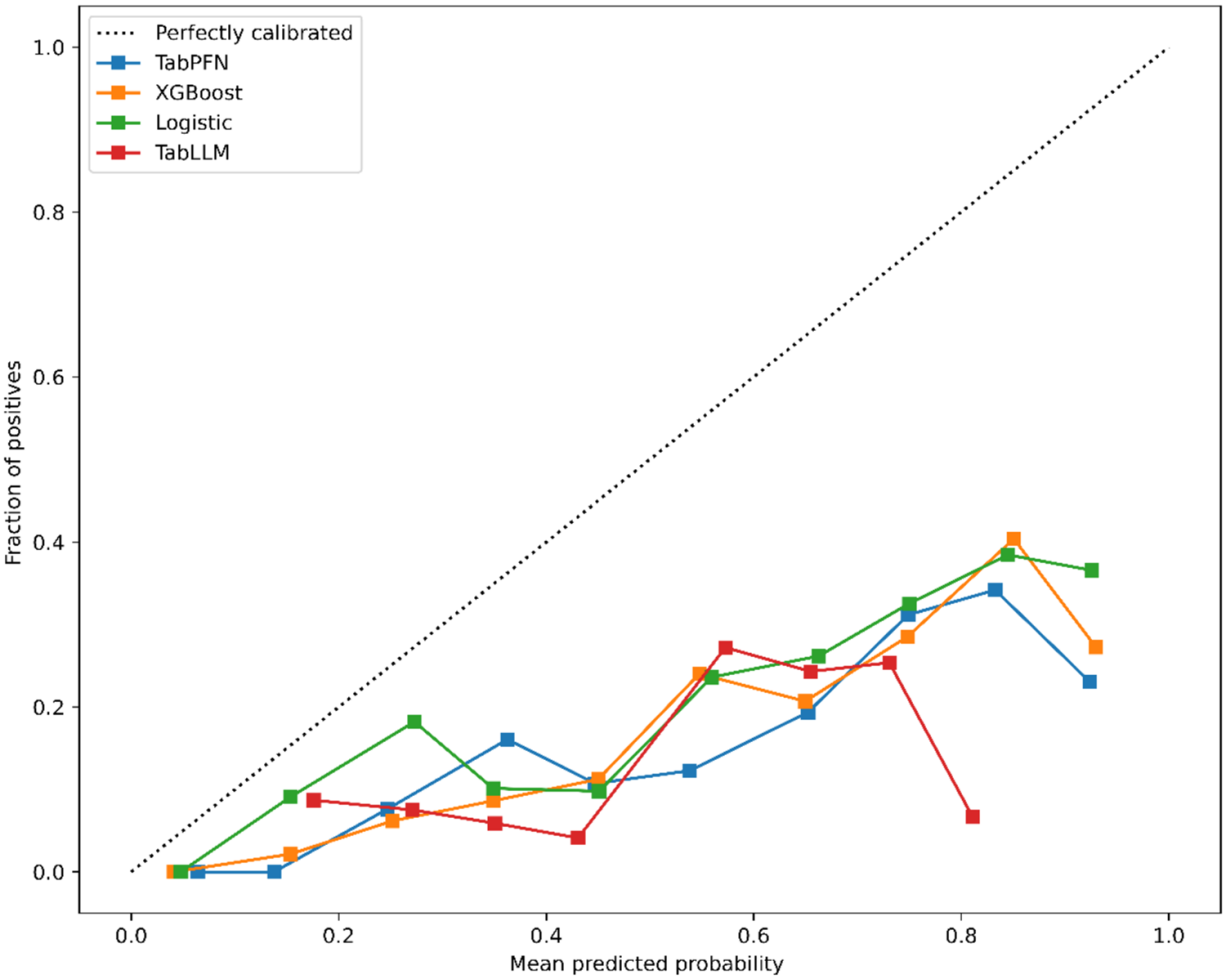
Models calibration. The black dotted line represents perfect calibration. The blue line is the TabPFN model. The yellow line is the GB model. The green line is the LR model. The red line is the TabLLM model

The GB model achieved the highest AUC of 0.78, followed by the LR model with an AUC of 0.75. According to the MCC metric, the GB model demonstrated the best performance, with a mean MCC of 0.32. The TabPFN and TabLLM model BS had the lowest values reaching 0.43 and 0.32, respectively. Model performance is reported in Table 3 (part A). ROC curves and calibration curves are shown in Figs. 2 and 3.

**Table 3:** Performance metrics (part A) and models statistical differences (part B)

A			
	AUC	MCC	BS
GB	0.78	0.32	0.46
LR	0.75	0.29	0.44
TabPFN	0.71	0.21	0.43
TabLLM	0.69	0.27	0.32

*GB* gradient boosting, *LR* logistic regression, *TabLLM* tabular large language model, *TabPFN* tabular prior-data fitted network, *AUC* area under the curve, *MCC* Matthews correlation coefficient, *BS* Brier score

The GB model performed significantly better than the TabLLM and TabPFN models but it was only slightly better than the LR model. Results of the paired *t*-test are presented in Table 3 (part B).

## Discussion

In the present study, the ML models developed by Grant et al. on a Canadian population [16] were tested on an external cohort of Italian syncope patients to assess their effectiveness in risk prediction. In addition, the performance of these models [16] was compared with that of novel DL algorithms.

We found that the classic ML model GB, achieved the highest AUC values. Moreover, these traditional ML models outperformed the recently developed DL models utilized in this study for syncope risk prediction.

The results of our study indicate that the prediction capability of the previously established models GB and LR [16] decreased when validated on the current cohort of syncope patients, as evidenced by lower AUC values (0.78 and 0.75, respectively) compared to the original results (0.91 and 0.90, respectively). Several points should be considered in interpreting these findings, as the Italian external validation cohort in the current study significantly differed from the original derivation cohort [16].

Firstly, the Italian validation cohort comprised individuals presenting with syncope at six different EDs, all characterized as non-low risk based on the assessment of the physician in charge [26]. According to guidelines and position papers [2–6], because of the non-low- risk profile, syncope patients should undergo further, detailed evaluation for appropriate management, which might include clinical observation with continuous monitoring in the ED [3, 27, 28], hospital admission to an adequate ward with medium-term monitoring facilities, or fast-track referral to a Syncope Unit for expedited outpatient assessment [29]. Given the complexity of a clinical decision making in this context, we reasoned that selecting a non-low-risk population would provide the most suitable cohort to test the effectiveness and practical utility of Grant et al. risk stratification algorithms [16]. Not surprisingly, in our non-low-risk population, the number of adverse outcomes was found to be greater than that of the Canadian population, with a rate of events occurring in 13% compared to 3.6% in the Canadian derivation cohort [16]. This discrepancy can also be partially explained by differences between healthcare systems and local syncope management protocols in the two countries, such as the common practice of immediate ED discharge for low-risk syncope patients in Italian hospitals. Lastly, the mean age of the Italian validation cohort was higher than the original Canadian derivation cohort, with a median age of 71 years compared to a mean of 54 years in the Canadian training and test sets [16].

Taken together, these differences indicate substantial variations in the case mix between the two cohorts, likely explaining the observed discrepancies in predictive performance of the original models in the current investigation.

Novel risk stratification tools should take into account differences in healthcare systems where they are implemented. Over-diagnosis in resource-limited settings may be counterproductive, as these systems might lack the capacity to manage a high volume of false positives. This could lead to unnecessary investigations, increased strain on limited resources, and potentially overlooking other critical conditions. The implementation of AI could enhance efficiency and diagnostic accuracy without further burdening already stressed healthcare systems.

The CSRS was recently proposed as a new decision rule for syncope risk stratification, showing high sensitivity and accuracy within a Canadian clinical setting. We recently validated CSRS on an Italian syncope cohort using classical statistics and found its predictive accuracy to be similar to clinical judgement [29]. In addition, the latter would have enabled the discharge from the ED of fewer patients suffering from 30-day adverse events compared with CSRS.

In the present study, we employed the CSRS predictors with novel algorithms: a DL model, TabPFN, and a large language-based model, TabLLM, to assess their prediction capability on a multicentre Italian clinical dataset. Notably, both TabPFN and TabLLM models were previously developed and used to improve and solve classification problems, particularly the extraction of medical information from tabular data [30].

The results of the current study suggest that both TabPFN and TabLLM models underperformed compared to the classic ML-based models, such as GB, and to LR [16]. A possible explanation for this limitation may relate to the tabular attributes of our dataset largely comprehending binary data. The latter is known to bear limited amount of information compared to numerical data. Supporting this hypothesis, age emerged as a significant predictor of adverse outcomes according to the GB model. Notably, age was numerical data within the validation dataset and therefore likely conveyed more substantial information compared to simple binary (yes/no) data. These aspects, combined with the limited size of our study population, might have impaired the effectiveness of both TabPFN and TabLLM algorithms in accurately extracting medical information for a proper syncope risk stratification [30, 31].

Compared to other risk stratification tools, the algorithms presented in this study achieved a numerically lower AUC. In their respective validation studies, the OESIL score reported an AUC of 0.89, the EGYS score 0.80, and the SFSR 0.92 [11, 31–34]. However, several important differences should be noted. First, the mean age in the present study population was higher, indicating a more fragile cohort potentially subject to multiple concurrent factors contributing to adverse events. Second, the algorithms developed in this study utilized a greater number of predictors compared to the EGYS, SFSR, and OESIL scores, which may reduce their generalizability. Finally, the predictors used in our model included specific ECG criteria that differed from those used in the other scores, which could also have influenced the algorithm’s overall performance.

### Limitations

We acknowledge a few limitations of the current study. Although based on a multicenter dataset, our validation cohort consisted mostly of data obtained from a relatively small number of patients, which might have affected the performance of Grant et al. models [16]. However, as highlighted in a recent publication [17], homogeneous and focused datasets, such as the one used in our study, are likely to improve data accuracy and yield reliable AI-based prediction even with limited sample sizes. In the present study, specifically using a non-low-risk syncope cohort for validation purposes likely led to substantial differences in the case mix between the Canadian derivation population and our Italian validation population, potentially impacting the predictive effectiveness of the Grant et al. models [16]. This further underscores that a syncope risk model developed from one specific population may not be easily generalizable to other syncope cohorts without affecting the model’s prediction capability.

The role of troponin as a predictor in this investigation deserves additional commentary. Troponin values frequently were missing in our validation dataset. Given that troponin is a highly specific marker for myocardial ischemia, it is reasonable to hypothesize that ED physicians ordered this test only when an ischemic cause of syncope was strongly suspected, consistent with guideline recommendations [2, 3] and a Choosing Wisely approach [32, 33]. We would like to point out that we tested three different methods for automatic troponin data imputation (SimpleImputer, IterativeImpuiter and KNNInputer) and found no significant differences in the GB model’s performance. Thus, given the high number of missing values, troponin’s utility as a reliable predictor of adverse outcomes in this context is limited. In contrast, other variables such as QRS axis and age, which were included in the GB model but not in the LR model, may have contributed to the superior predictive effectiveness observed for the GB model in our study. Indeed, these variables are usually available for every syncope patient. Moreover, an abnormal QRS axis is a strong indicator of cardiac disease, aiding in the identification of patients with underlying structural heart disease [34].

It is important to point out that in order to make models understandable, SHAP and LIME methodologies might have been used effectively. However, since the current study aim was to validate a previously set model, it seemed out of the scope of the present investigation to address that issue. Following this line of thought, also potential novel predictors were not added.

## Conclusions

The results of the current study indicate that the prediction capability of previously developed ML models [16] decreased when validated on an external cohort of non-low-risk syncope patients. Furthermore, the GB-based model outperformed more recently developed DL models developed in this study for predicting severe syncope-related outcomes.

## Data Availability

The data underlying this article can be shared on reasonable request to the corresponding author.

## Abbreviations

AI: Artificial intelligence
AUC: Area under the curve
BERT: Bidirectional encoder representations from transformers
CSRS: Canadian syncope risk score
ECG: Electrocardiogram
ED: Emergency department
GB: Gradient boosting
ICD: Implantable cardioverter defibrillator
LR: Logistic regression
MCC: Matthews correlation coefficient
ML: Machine learning
PM: Permanent pacemaker
ROC: Receiver operating characteristic
TabLLM: Tabular large language model
TabPFN: Tabular prior-data fitted network

## Declarations

### Human and animal right

The study was approved by the institutional ethics committee. This article does not contain any studies with human participants or animals performed by any of the authors.

## References

[1] Furlan L, Jacobitti Esposito G, Gianni F, Solbiati M, Mancusi C, Costantino G (2024) Syncope in the emergency department: a practical approach. J Clin Med. 13:3231. 10.3390/JCM13113231 38892942 10.3390/jcm13113231 PMC11172976

[2] Shen WK, Sheldon RS, Benditt DG, Cohen MI, Forman DE, Goldberger ZD et al (2017) 2017 ACC/AHA/HRS guideline for the evaluation and management of patients with syncope: a report of the American College of Cardiology/American Heart Association Task Force on Clinical Practice Guidelines and the Heart Rhythm Society. J Am Coll Cardiol 70:e39-110. 10.1016/J.JACC.2017.03.003 28286221 10.1016/j.jacc.2017.03.003

[3] Brignole M, Moya A, De Lange FJ, Deharo JC, Elliott PM, Fanciulli A et al (2018) 2018 ESC guidelines for the diagnosis and management of syncope. Eur Heart J 39:1883–1948. 10.1093/EURHEARTJ/EHY037 29562304 10.1093/eurheartj/ehy037

[4] Costantino G, Sun BC, Barbic F, Bossi I, Casazza G, Dipaola F et al (2015) Syncope clinical management in the emergency department: a consensus from the first international workshop on syncope risk stratification in the emergency department. Eur Heart J 37:1493. 10.1093/EURHEARTJ/EHV378 26242712 10.1093/eurheartj/ehv378 PMC4872282

[5] Grossman SA, Fischer C, Lipsitz LA, Mottley L, Sands K, Thompson S et al (2007) Predicting adverse outcomes in syncope. J Emerg Med 33:233–239. 10.1016/J.JEMERMED.2007.04.001 17976548 10.1016/j.jemermed.2007.04.001 PMC2276584

[6] Reed MJ, Newby DE, Coull AJ, Prescott RJ, Jacques KG, Gray AJ (2010) The ROSE (risk stratification of syncope in the emergency department) study. J Am Coll Cardiol 55:713–721. 10.1016/J.JACC.2009.09.049 20170806 10.1016/j.jacc.2009.09.049

[7] Thiruganasambandamoorthy V, Kwong K, Wells GA, Sivilotti MLA, Mukarram M, Rowe BH et al (2016) Development of the Canadian Syncope Risk Score to predict serious adverse events after emergency department assessment of syncope. CMAJ 188:E289–E298. 10.1503/CMAJ.151469/-/DC1 27378464 10.1503/cmaj.151469 PMC5008955

[8] Martin TP, Hanusa BH, Kapoor WN (1997) Risk stratification of patients with syncope. Ann Emerg Med 29:459–466. 10.1016/S0196-0644(97)70217-8 9095005 10.1016/s0196-0644(97)70217-8

[9] Quinn JV, Stiell IG, McDermott DA, Sellers KL, Kohn MA, Wells GA (2004) Derivation of the San Francisco syncope rule to predict patients with short-term serious outcomes. Ann Emerg Med 43:224–232. 10.1016/S0196-0644(03)00823-0 14747812 10.1016/s0196-0644(03)00823-0

[10] Del Rosso A, Ungar A, Maggi R, Giada F, Petix NR, De Santo T et al (2008) Clinical predictors of cardiac syncope at initial evaluation in patients referred urgently to a general hospital: the EGSYS score. Heart 94:1620–1626. 10.1136/HRT.2008.143123 18519550 10.1136/hrt.2008.143123

[11] Colivicchi F, Ammirati F, Melina D, Guido V, Imperoli G, Santini M (2003) Development and prospective validation of a risk stratification system for patients with syncope in the emergency department: the OESIL risk score. Eur Heart J 24:811–819. 10.1016/S0195-668X(02)00827-8 12727148 10.1016/s0195-668x(02)00827-8

[12] Costantino G, Casazza G, Reed M, Bossi I, Sun B, Del Rosso A et al (2014) Syncope risk stratification tools vs clinical judgment: an individual patient data meta-analysis. Am J Med 127:1126.e13-1126.e25. 10.1016/J.AMJMED.2014.05.022 24862309 10.1016/j.amjmed.2014.05.022

[13] Costantino G, Falavigna G, Solbiati M, Casagranda I, Sun BC, Grossman SA et al (2017) Neural networks as a tool to predict syncope risk in the emergency department. EP Europace 19:1891–1895. 10.1093/EUROPACE/EUW336 10.1093/europace/euw336 28017935

[14] Solbiati M, Quinn JV, Dipaola F, Duca P, Furlan R, Montano N et al (2020) Personalized risk stratification through attribute matching for clinical decision making in clinical conditions with aspecific symptoms: the example of syncope. PLoS One. 10.1371/JOURNAL.PONE.0228725 32187195 10.1371/journal.pone.0228725 PMC7080223

[15] Dipaola F, Gatti M, Menè R, Shiffer D, Giaj Levra A, Solbiati M et al (2023) A hybrid model for 30-day syncope prognosis prediction in the emergency department. J Pers Med 14:4. 10.3390/JPM14010004 38276219 10.3390/jpm14010004 PMC10817569

[16] Grant L, Joo P, Nemnom MJ, Thiruganasambandamoorthy V (2022) Machine learning versus traditional methods for the development of risk stratification scores: a case study using original Canadian Syncope Risk Score data. Intern Emerg Med 17:1145–1153. 10.1007/S11739-021-02873-Y 34734350 10.1007/s11739-021-02873-y

[17] Dipaola F, Gebska MA, Gatti M, Levra AG, Parker WH, Menè R et al (2024) Will artificial intelligence be “Better” than humans in the management of syncope? JACC Adv. 10.1016/J.JACADV.2024.101072 39372450 10.1016/j.jacadv.2024.101072 PMC11450913

[18] Solbiati M, Dipaola F, Villa P, Seghezzi S, Casagranda I, Rabajoli F et al (2020) Predictive accuracy of electrocardiographic monitoring of patients with syncope in the emergency department: the SyMoNE multicenter study. Acad Emerg Med 27:15–23. 10.1111/ACEM.13842 31854141 10.1111/acem.13842

[19] Sun BC, Thiruganasambandamoorthy V, Dela CJ (2012) Standardized reporting guidelines for emergency department syncope risk-stratification research. Acad Emerg Med 19:694–702. 10.1111/J.1553-2712.2012.01375.X 22687184 10.1111/j.1553-2712.2012.01375.x PMC3376009

[20] Huang K, Altosaar J, Ranganath R. ClinicalBERT: Modeling Clinical Notes and Predicting Hospital Readmission 2019.

[21] GitHub—automl/TabPFN: Official implementation of the TabPFN paper (https://arxiv.org/abs/2207.01848) and the tabpfn package. n.d. https://github.com/automl/TabPFN (accessed July 9, 2024).

[22] Hollmann N, Müller S, Eggensperger K, Hutter F. TabPFN: a transformer that solves small tabular classification problems in a second 2022.

[23] Hegselmann S, Buendia A, Lang H, Agrawal M, Jiang X, Sontag D (2022) TabLLM: few-shot classification of tabular data with large language models. Proc Mach Learn Res 206:5549–5581

[24] Chicco D, Warrens MJ, Jurman G (2021) The Matthews Correlation Coefficient (MCC) is more informative than Cohen’s Kappa and Brier score in binary classification assessment. IEEE Access 9:78368–78381. 10.1109/ACCESS.2021.3084050

[25] Chicco D, Jurman G (2020) The advantages of the Matthews correlation coefficient (MCC) over F1 score and accuracy in binary classification evaluation. BMC Genomics 21:1–13. 10.1186/S12864-019-6413-7/TABLES/5 10.1186/s12864-019-6413-7 PMC6941312 31898477

[26] Furlan L, Trombetta L, Casazza G, Dipaola F, Furlan R, Marta C et al (2021) Syncope time frames for adverse events after emergency department presentation: an individual patient data meta-analysis. Medicina (Lithuania) 57:1235. 10.3390/MEDICINA57111235/S1 10.3390/medicina57111235 PMC8623370 34833453

[27] Solbiati M, Trombetta L, Sacco RM, Erba L, Bozzano V, Costantino G et al (2019) A systematic review of noninvasive electrocardiogram monitoring devices for the evaluation of suspected cardiovascular syncope. J Med Dev Trans ASME. 10.1115/1.4042795/368681

[28] Thiruganasambandamoorthy V, Rowe BH, Sivilotti MLA, McRae AD, Arcot K, Nemnom MJ et al (2019) Duration of electrocardiographic monitoring of emergency department patients with syncope. Circulation 139:1396–1406. 10.1161/CIRCULATIONAHA.118.036088/SUPPL_FILE/CIRC_CIRCULATIONAHA-2018-036088_SUPP1.PDF 30661373 10.1161/CIRCULATIONAHA.118.036088

[29] Kenny RA, Brignole M, Dan GA, Deharo JC, Van Dijk JG, Doherty C et al (2015) Syncope unit: rationale and requirement–the European Heart Rhythm Association position statement endorsed by the Heart Rhythm Society. Europace 17:1325–1340. 10.1093/EUROPACE/EUV115 26108809 10.1093/europace/euv115

[30] Agrawal M, Hegselmann S, Lang H, Kim Y, Sontag D. Large Language Models are Few-Shot Clinical Information Extractors. Proceedings of the 2022 Conference on Empirical Methods in Natural Language Processing, EMNLP 2022 2022:1998–2022. 10.18653/v1/2022.emnlp-main.130.

[31] Liu S-Y, Ye H-J. TabPFN unleashed: a scalable and effective solution to tabular classification problems 2025.

[32] Furlan L, Di FP, Costantino G, Montano N (2022) Choosing Wisely in clinical practice: embracing critical thinking, striving for safer care. J Intern Med 291:397–407. 10.1111/JOIM.13472 35307902 10.1111/joim.13472 PMC9314697

[33] Costantino G, Furlan L, Bracco C, Cappellini MD, Casazza G, Nunziata V et al (2021) Impact of implementing a choosing wisely educational intervention into clinical practice: the CW-SIMI study (a multicenter-controlled study). Eur J Intern Med 93:71–77. 10.1016/J.EJIM.2021.07.008 34353705 10.1016/j.ejim.2021.07.008

[34] Costantino G, Perego F, Dipaola F, Borella M, Galli A, Cantoni G et al (2008) Short- and long-term prognosis of syncope, risk factors, and role of hospital admission: results from the STePS (Short-Term Prognosis of Syncope) study. J Am Coll Cardiol 51:276–283. 10.1016/J.JACC.2007.08.059 18206736 10.1016/j.jacc.2007.08.059

